# Clinical indicators for dose re-optimization in magnetic resonance-guided online adaptive radiotherapy for prostate cancer

**DOI:** 10.1016/j.phro.2026.101060

**Published:** 2026-08-05

**Authors:** Masato Nishitani, Hiroyuki Okamoto, Weishan Chang, Junichi Kuwahara, Takahito Chiba, Tatsuya Sakasai, Hiroki Nakayama, Koji Inaba, Tairo Kashihara, Satoshi Nakamura, Tetsu Nakaichi, Tomonori Goka, Hiroshi Igaki

**Affiliations:** a Department of Radiological Sciences, Graduate School of Human Health Sciences, Tokyo Metropolitan University, Higashi-ogu 7-2-10, Arakawa-ku, Tokyo 116-8551, Japan; b Section of Radiation Safety and Quality Assurance, National Cancer Center Hospital, Tsukiji 5-1-1, Chuo-ku, Tokyo 104-0045, Japan; c Department of Radiological Technology (Radiological Oncology), National Cancer Center Hospital, Tsukiji 5-1-1, Chuo-ku, Tokyo 104-0045, Japan; d Department of Radiation Oncology, National Cancer Center Hospital, Tsukiji 5-1-1, Chuo-ku, Tokyo 104-0045, Japan; e Department of Radiological Sciences, Nagasaki University Graduate School of Biomedical Sciences, Nagasaki, Japan

**Keywords:** MR-guided online adaptive radiotherapy, Treatment planning analysis, Clinical indicators for dose re-optimization

## Abstract

**Background and Purpose:**

This study aimed to identify dose-volume parameters and anatomical features associated with the implementation of online dose re-optimization in magnetic resonance-guided adaptive radiotherapy (ART) for prostate cancer and to evaluate its impact on target coverage and organs-at-risk dose-volume parameters.

**Materials and Methods:**

Treatment plans from 150 fractions (with and without dose re-optimization) in 30 patients receiving five-fraction stereotactic body radiotherapy were retrospectively analyzed. Evaluated parameters included planning target volume (PTV) *V*_100%_, bladder and rectum dose-volume parameters, volumetric changes, and relationship between changes in target coverage.

**Results:**

Online dose re-optimization was performed in 60% of fractions and became more frequent in later fractions (*p* = 0.02). Compared with reference plans, non-ART plans showed a 3.7% lower median PTV *V*_100%_ (*p* < 0.01), and 65.3% of fractions failed to achieve the 95% coverage threshold. The target volume increased significantly after the third fraction (*p* < 0.05). Target volume expansion was associated with reductions in PTV *V*_100%_ in non-ART plans, while this relationship was substantially weakened after dose re-optimization. Although bladder and rectum dose-volume parameters increased significantly in non-ART plans, all median values remained within predefined dose constraints.

**Conclusions:**

Progressive target-volume expansion appears to be the primary anatomical factor associated with deterioration in target coverage. A reduction in PTV *V*_100%_, particularly below 95%, represented a clinically meaningful indicator for online dose re-optimization. These findings suggested that online dose re-optimization remained important for maintaining target coverage and limiting gradual deterioration of organs-at-risk dose-volume parameters, even when predicted plans satisfied clinical constraints.

## Highlights

• More than half of the non-adaptive radiotherapy fractions (65.3%) showed planning target volume *V*_100%_ < 95%.
• Without re-optimization, median target coverage decreased by 3.7%.
• Target-volume expansion correlated with reduced target coverage (*r* = −0.65 to −0.37).

## Graphical abstract

**Figure:**
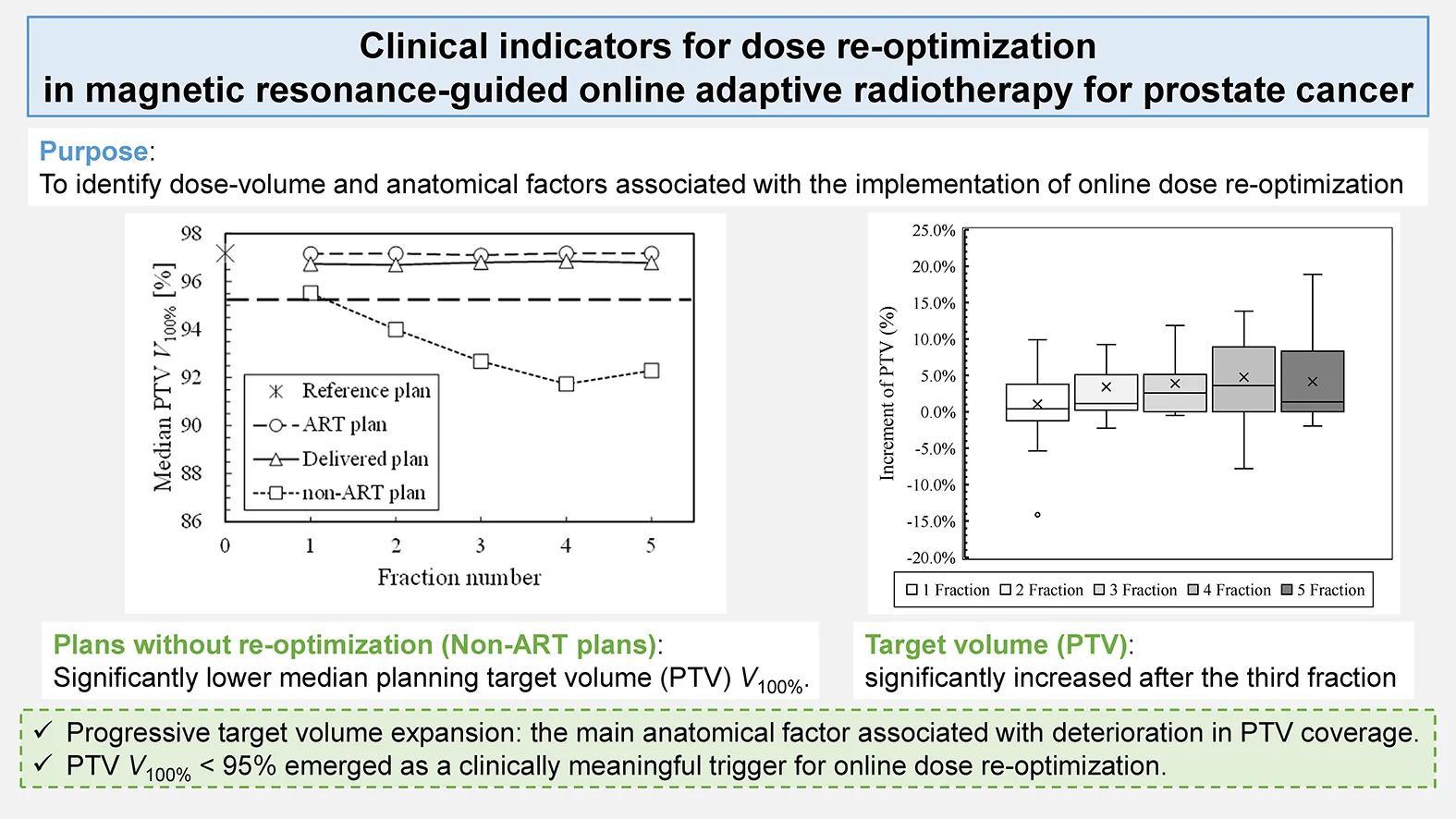

## 1 Introduction

The integration of magnetic resonance (MR) imaging system and a linear accelerator (Linac) has recently enabled the clinical implementation of MR-guided radiotherapy [1]. This technological advancement has not only facilitated the use of MR images with superior soft tissue contrast for treatment planning and patient positioning but has also supported the development of personalized and adaptive radiotherapy strategies. Unlike traditional offline adaptive radiotherapy, which may require additional planning time, MR-guided radiotherapy enables online adaptive radiotherapy (online ART) [2], [3], [4], [5]. Online ART involves the modification of target and organs at risk (OAR) contours and dose re-optimization while the patient remains in the treatment position. This workflow ensures that the planned dose is delivered as accurately as possible according to the patient's anatomy at each treatment fraction.

MR-guided online ART offers significant benefits for prostate cancer, as the clear visualization of the boundary between the prostate and surrounding soft tissues reduces inter-observer variability in contouring and improves the accuracy of delineation [6], [7], [8]. Furthermore, even with accurate initial delineation, substantial inter-fractional variations in bladder and rectal filling compared to the initial planning images may still affect dose delivery. This underscores the added importance of online ART in managing daily anatomical changes, especially through dose re-optimization [4].

While online ART enables personalized treatment based on the MR images for precise patient alignment prior to irradiation, the dose re-optimization and dose-volumetric evaluation process, in particular, can take approximately 10 to 30 min, presenting potential challenges for clinical workflow efficiency [9]. Therefore, establishing clear criteria for determining when to perform online ART can help reduce treatment time and improve workflow efficiency.

When using a 0.35 T MR-Linac system, the decision to perform online dose re-optimization is often primarily based on deviations from dose constraints, such as OAR dose tolerances [10]. In contrast, a commercially available 1.5 T MR-Linac system mandates dose re-optimization for every treatment fraction. A 0.35 T MR-Linac system provides flexibility to determine whether online dose re-optimization should be performed following contour modifications and dose evaluation [11]. However, there is no standardized guideline for deciding when to apply online ART in prostate cancer and relying solely on dose constraint compliance may not ensure optimal dosimetry. Several previous studies have reported the clinical feasibility and dose-volume parameters advantages of online dose re-optimization using MR-linac systems, highlighting improvements in target coverage and OAR sparing [12], [13]. Despite this, clear objective criteria guiding the decision to perform online dose re-optimization in prostate cancer remain insufficiently defined. Further investigation is therefore needed to identify appropriate indicators for dose re-optimization.

Furthermore, other previous studies have reported that prostate volume and rotation tend to increase as treatment progresses [14], [15], [16], [17], [18], which may be associated with the need for online dose re-optimization and can serve as a contributing factor for its implementation. The aim of this study is to identify anatomical and dose-volume characteristics associated with the implementation of dose re-optimization and to explore clinically relevant indicators that can guide its use in prostate cancer treatment.

## 2 Materials and methods

### 2.1 Patient's characteristics

This retrospective study was approved by the Institutional Review Board of the National Cancer Center Hospital (Approval No. 2017–091). Our study included 30 prostate cancer patients who underwent 5 fraction stereotactic body radiotherapy with the MRIdian Linac (ViewRay Systems Inc. Oakwood Village, OH, USA) between August 2022 and June 2023. Table 1 summarizes the patient's age, the value of prostate specific antigen (PSA), prostate volume in reference plan, number of patients in the prostate cancer risk groups based on NCCN Guidelines [19], total prescribed dose, inclusion of the seminal vesicle in the planning target volume (PTV), and hydrogel spacer insertion. The median age (range: minimum–maximum) and the value of PSA were 69 (47–85) and 9.5 (3.9–117.5) ng/mL, respectively. A hydrogel spacer [20] was implanted in 14 out of 30 patients.

**Table 1:** Patient characteristics and treatment information.

	Median (minimum - maximum)
Age	69 (47–85)
PSA	9.5 (3.9–117.5) ng/mL
Prostate volume in reference plan	24.5 (10.6–49.3)
	Number of patients (%)

Risk group	
Very low	1 (3.3)
Intermediate	20 (66.7)
High	5 (16.7)
Very high	4 (13.3)

Total prescribed dose	
36.25 Gy	16 (53.3)
37.50 Gy	12 (40.0)
40.00 Gy	2 (6.7)

Inclusion of the seminal vesicle in the PTV	
Yes / No	14 (46.7) / 16 (53.3)

Hydrogel spacer insertion	
Yes / No	14 (46.7) / 16 (53.3)

### 2.2 Treatment procedure

Computed tomography (CT) and MR images were acquired on the same day, and bladder filling was managed according to institutional protocols (approximately 200 cm^3^). The clinical target volume (CTV) and OAR were delineated, and the PTV was generated using anisotropic margins (3 mm posteriorly, 5 mm laterally, and 4 mm in all other directions). For each fraction, daily MR images were acquired, and contours were revised as necessary. The reference plan was recalculated on the updated anatomy and assessed using the dose constraints summarized in Table 2 [10], while the decision to perform online ART was not based solely on constraint compliance. The final decision was made by the radiation oncologists after consideration of target coverage, dose distribution, OAR dose parameters, and overall clinical judgment.

**Table 2:** Dose constraints considered in our study [10].

Dose targets	Constraints
PTV	*V*_100%_ ≧ 95%
Bladder	*V*_37 Gy_ < 5 cm^3^
	*V*_100%_ < 10%
	*V*_50%_ < 40%
Rectum	*V*_36 Gy_ < 1 cm^3^
	*V*_100%_ < 5%
	*V*_90%_ < 10%
	*V*_80%_ < 20%
	*V*_75%_ < 25%
	*V*_50%_ < 50%

### 2.3 Proportion of treatment fractions with dose re-optimization

Given that anatomical variations can lead to fluctuations in compliance with dose constraints, we analyzed the proportion of treatment fractions requiring online dose re-optimization. Differences in dose re-optimization frequency between treatment fractions were evaluated using the chi-square test. Furthermore, patients were stratified into subgroups to explore clinical and anatomical factors potentially influencing the decision to implement online ART. First, patients were divided into two groups based on prescription dose (36.25 Gy vs. ≥ 37.50 Gy), as maintaining adequate target coverage and adherence to dose constraints becomes increasingly challenging with higher prescription doses, despite the application of identical OAR dose constraints across all patients. Second, to assess the potential impact of seminal vesicle motion on the decision to perform online ART, patients were stratified according to whether the seminal vesicles were included in the PTV in each treatment plan [21]. Third, given the potential influence of rectum anatomy stabilization on prostate motion and dose-volume parameters robustness, patients were additionally stratified based on the presence or absence of a hydrogel spacer. For each subgroup, the fraction-wise implementation rate of online ART was evaluated.

### 2.4 Survey of potential characteristics of online ART implementation

**Fig. 1:**
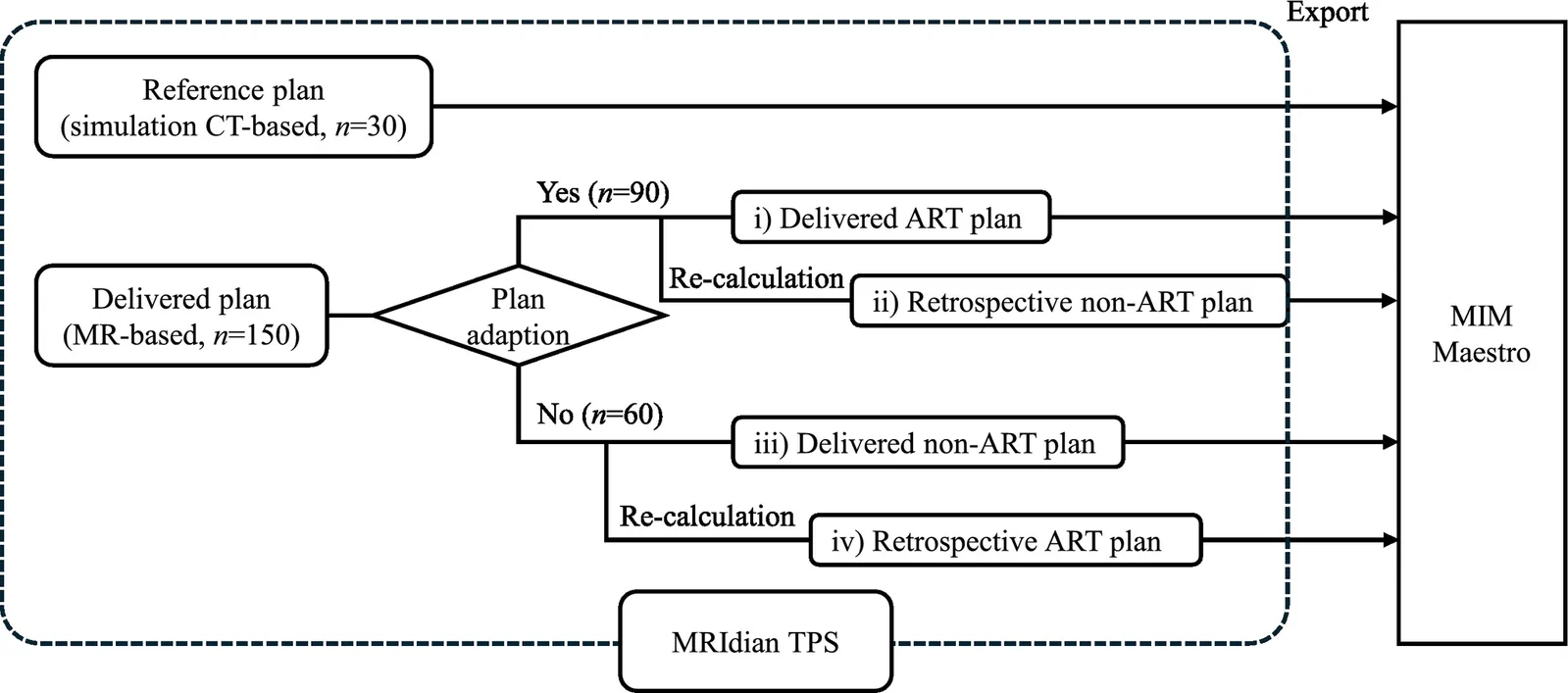
Flowchart of the analysis process. Reference CT-based reference plans (*n* = 30 patients) and 150 actual delivered plans (MR-based) were analyzed. The 150 fractions were categorized into adapted (*n* = 90) and non-adapted (*n* = 60) groups. For each group, in addition to the actual delivered plans, we retrospectively generated the corresponding adapted or non-adapted plans for analysis.

Fig. 1 shows flowchart of the analysis process used in this study. For fractions in which online dose re-optimization was clinically performed, both (i) delivered ART plan and (ii) retrospective non-ART plan generated before dose re-optimization in the MRIdian treatment planning system (TPS) were exported to MIM Maestro (Version 7.1.4, MIM Software Inc., Cleveland, OH, USA). For fractions in which online dose re-optimization was not clinically performed, both (iii) delivered non-ART plan and (iv) retrospective ART plan generated by dose re-optimizing the treatment plan using the same-day MR images in the MRIdian TPS were exported to MIM Maestro. Plans in categories (i) and (iv) were defined as “ART plans”, whereas plans in categories (ii) and (iii) were defined as “non-ART plans”.

Dose-volume parameters for the PTV, bladder, and rectum were evaluated for the reference, delivered, ART, and non-ART plans using the criteria of Chen et al. [10] (Table 2). Urethra constraints were not evaluated because they were not included in these criteria. The Wilcoxon signed-rank test was used to compare the reference plan and the non-ART plan to assess the dose distribution that would result from delivering the reference treatment plan without online dose re-optimization.

Additionally, differences in PTV *V*_100%_ for each fraction, as well as PTV contour similarity -evaluated using the dice similarity coefficient (*DSC*), volume and distance - assessed using the mean distance to agreement (*MDA*), were investigated at each fraction based on the reference plan using MIM Maestro [22]. To further investigate the relationship between anatomical volume changes and target coverage, correlation analyses were performed between the change in target size (*Δ*PTV) and the change in PTV *V*_100%_ (*Δ*PTV *V*_100%_) for each treatment fraction. Pearson's correlation coefficients were calculated separately for ART and non-ART plans to evaluate whether volumetric expansion was associated with deterioration in target coverage under each adaptive condition.

## 3 Results

Of the 150 delivered treatment plans (30 patients × 5 fractions) reviewed, 90 plans (60.0%) underwent online dose re-optimization, with each patient receiving a median of three re-optimized fractions. The implementation of online dose re-optimization increased from 40.0% in the first fraction to 70.0% in the fifth fraction (Fig. S1). Chi-square analysis revealed a significant difference in dose re-optimization rates between the first and fourth fraction (*p* = 0.04), and between the first and fifth fraction (*p* = 0.02).

Online dose re-optimization was more frequently performed in patients receiving ≥37.50 Gy than in those receiving 36.25 Gy (50.0–85.7% vs. 31.3–62.5% across fractions). Similar trends were observed for plans including the seminal vesicles within the PTV and for patients treated with a hydrogel spacer (Fig. S2 (a)-(c)). However, PTV *V*_100%_ did not differ significantly between the corresponding groups.

**Fig. 2:**
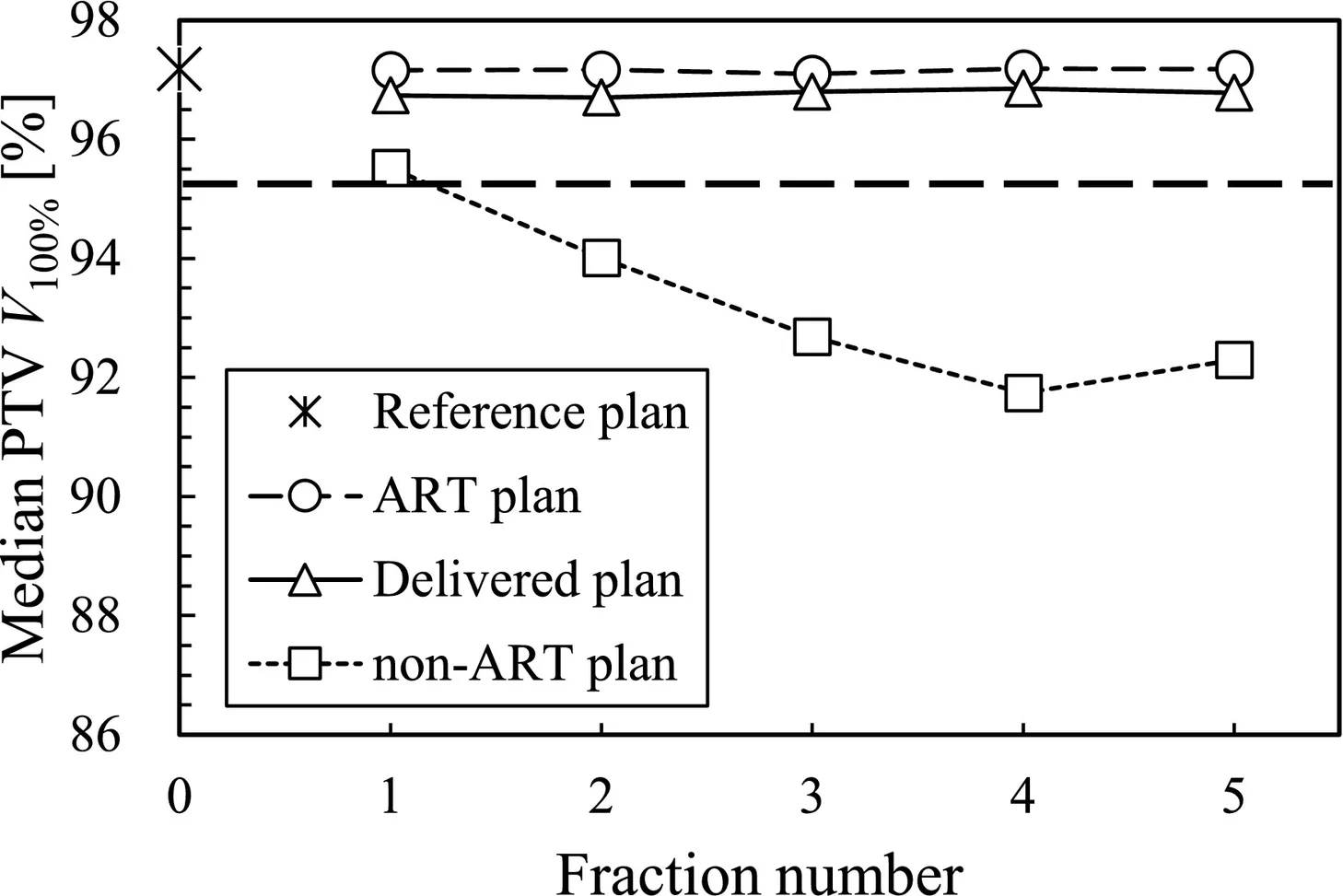
Median of PTV *V*_100%_ as a function of fraction number for the reference plan, delivered plan, ART plan and non-ART plan. The dashed line shows the dose constraint [10].

PTV *V*_100%_ exceeded 95% in 148 of 150 reference plans (98.7%). The median PTV *V*_100%_ in non-ART plans was 3.7% lower than that in reference plans (*p* < 0.01), and 65.3% of non-ART plans fell below the 95% coverage threshold. In contrast, all ART plans achieved PTV *V*_100%_ above 95% (Fig. S3). Fig. 2 shows the change in median PTV *V*_100%_ per fraction for the reference, delivered, ART and non-ART plans. Reference, delivered and ART plans maintained consistent target coverage (*V*_100%_ ≥ 95%) across all fractions, whereas the non-ART plans showed a 1.6–5.5% lower PTV *V*_100%_, indicating reduced target coverage compared with the ART plans across treatment fractions. A statistically significant difference was observed between the first and fifth fractions (ART vs. non-ART plans, *p* < 0.01).

Several bladder and rectum dose-volume parameters were significantly higher in the non-ART plans relative to the reference plans (Fig. S4 (a)-(e)). However, the median bladder *V*_100%_ was 2.5% in the ART plans and 2.2% in the non-ART plans, whereas the corresponding median rectum *V*_100%_ were 0.58% and 0.33%, respectively. All median bladder and rectum dose-volume parameters remained within the predefined dose constraints.

**Fig. 3:**
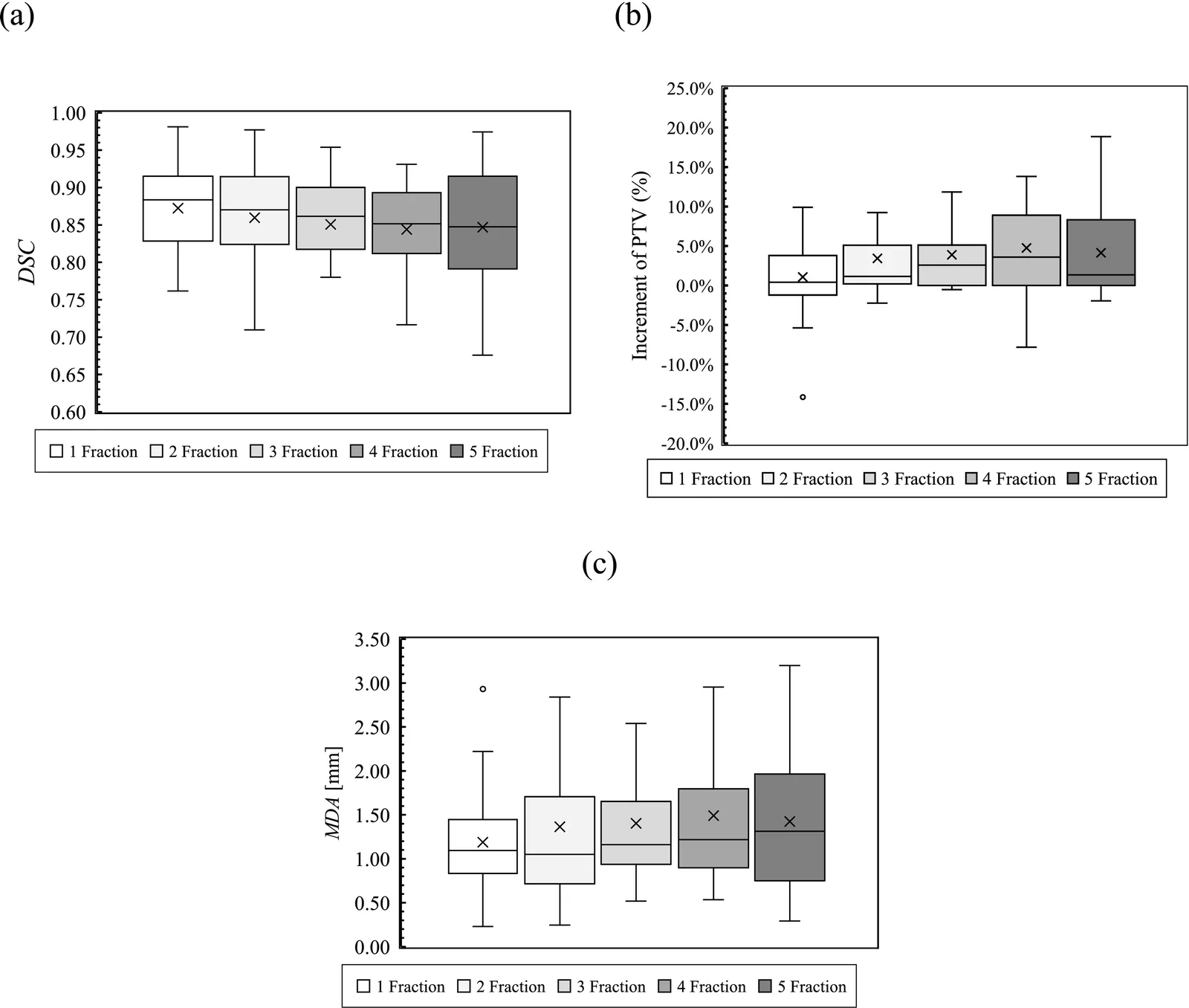
Boxplots of geometric and volumetric variations across treatment fractions. (a) *DSC* between reference plan's PTV and each fraction's PTV, (b) Increment of PTV from reference plan, (c) *MDA* between reference plan's PTV and each fraction's PTV. *DSC*: dice similarity coefficient, *MDA*: mean distance to agreement.

The geometric similarity between the reference PTV and the PTV at each treatment fraction was evaluated (Fig. 3). The median *DSC* gradually decreased over the treatment course; however, no statistically significant differences were observed between the first fraction and subsequent fractions, and the median *DSC* remained ≥0.85 throughout treatment (Fig. 3 (a)). The median PTV size increased from approximately 1.0% during the first two fractions to 2.6–3.6% after the third fraction (*p* < 0.05; Fig. 3 (b)). The median *MDA* also showed a gradual increasing trend with treatment progression, although the differences were not statistically significant (*p* > 0.05; Fig. 3 (c)). No statistically significant differences in PTV size were observed between prescription dose groups (36.25 Gy vs. ≥37.50 Gy). In addition to PTV, CTV also demonstrated a significant increase in volume (*p* < 0.05; Fig. S5).

Pearson's correlation coefficients between *Δ*PTV and *Δ*PTV *V*_100%_ ranged from −0.65 to −0.37 in non-ART plans, whereas correlation coefficients ranged from −0.29 to 0.19 in ART plans (Table 3).

**Table 3:** Pearson's correlation coefficient between *Δ*PTV and *Δ*PTV *V*_100%_ in non-ART plans for each fraction.

	Pearson's correlation coefficient	
Fraction	ART plan	non-ART plan
1	−0.29	−0.65
2	0.03	−0.37
3	−0.19	−0.53
4	−0.02	−0.60
5	0.19	−0.59

## 4 Discussion

In this study, online dose re-optimization was performed in 60% and became more frequent in later fractions. Without re-optimization, the median PTV *V*_100%_ decreased by 3.7%, and 65.3% of fractions fell below the clinical coverage threshold of 95%. Progressive target-volume expansion appeared to be associated with deterioration in target coverage. Furthermore, online dose re-optimization mitigated the impact of anatomical changes while maintaining OAR dose constraints.

The MRIdian-Linac allows fraction-by-fraction discretion in online dose re-optimization decisions, which in turn provides a unique opportunity to retrospectively compare adaptive and non-adaptive scenarios in a real-world clinical setting. While subgroup analyses demonstrated that higher prescription doses, seminal vesicles inclusion of the PTV, and hydrogel spacer placement were associated with more frequent online ART implementation (Fig. S2 (a)-(c)), the resulting PTV *V*_100%_ did not differ significantly between these subgroups. These findings suggest that radiation oncologists tended to implement online ART proactively in scenarios anticipated to be more vulnerable to anatomical variation or deterioration of dose-volume parameters, even before substantial reductions in target coverage became apparent.

A key finding of this study was the progressive deterioration of target coverage when online dose re-optimization was not performed (Fig. 2 and Fig. S3). Previous studies have consistently demonstrated that online ART improves target coverage in prostate cancer by accounting for daily anatomical variations [3], [4], [12], [13], [23], [24]. To elucidate the reasons underlying this deterioration, we examined both volumetric and positional changes. While the *DSC* gradually decreased across fractions, the *MDA* remained stable across all fractions (Fig. 3 (a), (c)), ruling out significant geometric shifts. Instead, significant increases in PTV size were observed post-fraction three (Fig. 3 (b), *p* < 0.05), suggesting that volumetric expansion contributed more substantially to coverage deterioration than positional displacement.

Pearson's correlation analysis demonstrated negative correlations between *Δ*PTV and *Δ*PTV *V*_100%_ in the non-ART plans, indicating that target-volume expansion was associated with deterioration in target coverage. In contrast, these correlations were substantially weakened in the ART plans, suggesting that online dose re-optimization effectively mitigated the impact of anatomical changes on dose-volume parameters [12], [25]. These findings extend previous reports demonstrating the benefits of online ART [12], [25] by further showing that target-volume expansion itself is closely associated with coverage deterioration when adaptation is not performed, whereas this relationship is largely eliminated after dose re-optimization. Consequently, longitudinal target-volume changes may serve as a clinically useful indicator for identifying fractions that are most likely to benefit from online dose re-optimization.

Previous studies have reported progressive prostate swelling during both conventionally fractionated and ultra-hypofractionated radiotherapy, with volume increases becoming more apparent during the later stages of treatment [15], [16], [17]. Our findings are consistent with these observations, as both CTV and PTV sizes increased significantly after the third fraction, while positional displacement remained stable. The median PTV size increment in our cohort increased to 2.6–3.6% after the third fraction, indicating a similar temporal pattern to previous reports. However, the magnitude of expansion was smaller than the approximately 8–15% prostate-volume increases reported in earlier studies [15], [16], [17], which may be attributable to differences in the evaluated structures (PTV versus prostate volume), contouring methodology, and fractionation schedules [26]. Furthermore, unlike previous studies that primarily focused on the overall benefits of online ART under daily anatomical variations [26], [27], the present study identified target-volume expansion as an anatomical factor associated with deterioration in PTV coverage when adaptation was not performed. The observed correlations between *Δ*PTV and *Δ*PTV *V*_100%_ suggest that volumetric expansion may represent one of the principal anatomical drivers of target coverage loss in MR-guided radiotherapy.

With respect to OAR dose-volume parameters, statistically significant differences were observed between reference and non-ART plans for bladder *V*_100%_, and rectum *V*_100%_ (Fig. S4 (a), (b)). Although median values remained within predefined dose constraints, the observed incremental increases suggest the presence of gradual volumetric drift across fractions. In hypofractionated regimens, even subtle systematic deviations may carry clinical implications. Online dose re-optimization may therefore serve not only as a corrective response to overt constraint violation, but also as a preventive strategy against progressive deterioration in both target coverage and OAR sparing.

Several limitations should be acknowledged. First, the present findings may depend on the specific PTV margins and dose-volume constraints used in our institutional workflow. Therefore, the proposed indicators for online dose re-optimization should be interpreted within the context of the treatment protocol applied in this study. Second, as a single-institution retrospective analysis with a modest cohort, this study lacks long-term clinical outcome data. Third, intra-fractional motion during the dose re-optimization process was not quantitatively evaluated, although real-time cine MRI monitoring with a 3 mm gating boundary was applied during treatment delivery. Future investigations incorporating predictive modeling of volumetric expansion and correlation with clinical outcomes may further refine objective criteria for adaptive decision-making.

In conclusion, this study found that progressive target-volume expansion represents an important anatomical factor associated with deterioration in target coverage during MR-guided online ART for prostate cancer. Without online dose re-optimization, PTV *V*_100%_ progressively declined across treatment fractions, indicating that coverage deterioration is a consequence of underlying anatomic evaluation. Therefore, monitoring target-volume expansion alongside PTV coverage may provide clinically meaningful indicators for determining when online dose re-optimization is warranted. Online dose re-optimization may thus serve both corrective and preventive roles in maintaining target coverage and limiting gradual OAR dose drift.

## CRediT authorship contribution statement

**Masato Nishitani:** Conceptualization. **Hiroyuki Okamoto:** Conceptualization. **Weishan Chang:** Data curation. **Junichi Kuwahara:** Funding acquisition. **Takahito Chiba:** Data curation. **Tatsuya Sakasai:** Funding acquisition. **Hiroki Nakayama:** Investigation. **Koji Inaba:** Investigation. **Tairo Kashihara:** Investigation. **Satoshi Nakamura:** Investigation. **Tetsu Nakaichi:** Investigation. **Tomonori Goka:** Investigation. **Hiroshi Igaki:** Project administration.

## Declaration of competing interest

The authors declare that they have no known competing financial interests or personal relationships that could have appeared to influence the work reported in this paper.
