# Supplementary material for: Clinical indicators for dose re-optimization in magnetic resonance-guided online adaptive radiotherapy for prostate cancer
Source: Phys Imaging Radiat Oncol. 2026 Aug 5;40:101060. doi: 10.1016/j.phro.2026.101060 (PMC13524625; doi:10.1016/j.phro.2026.101060)
Supplement: Supplementary file 1 [file mmc1.pdf]

Supplemental materials

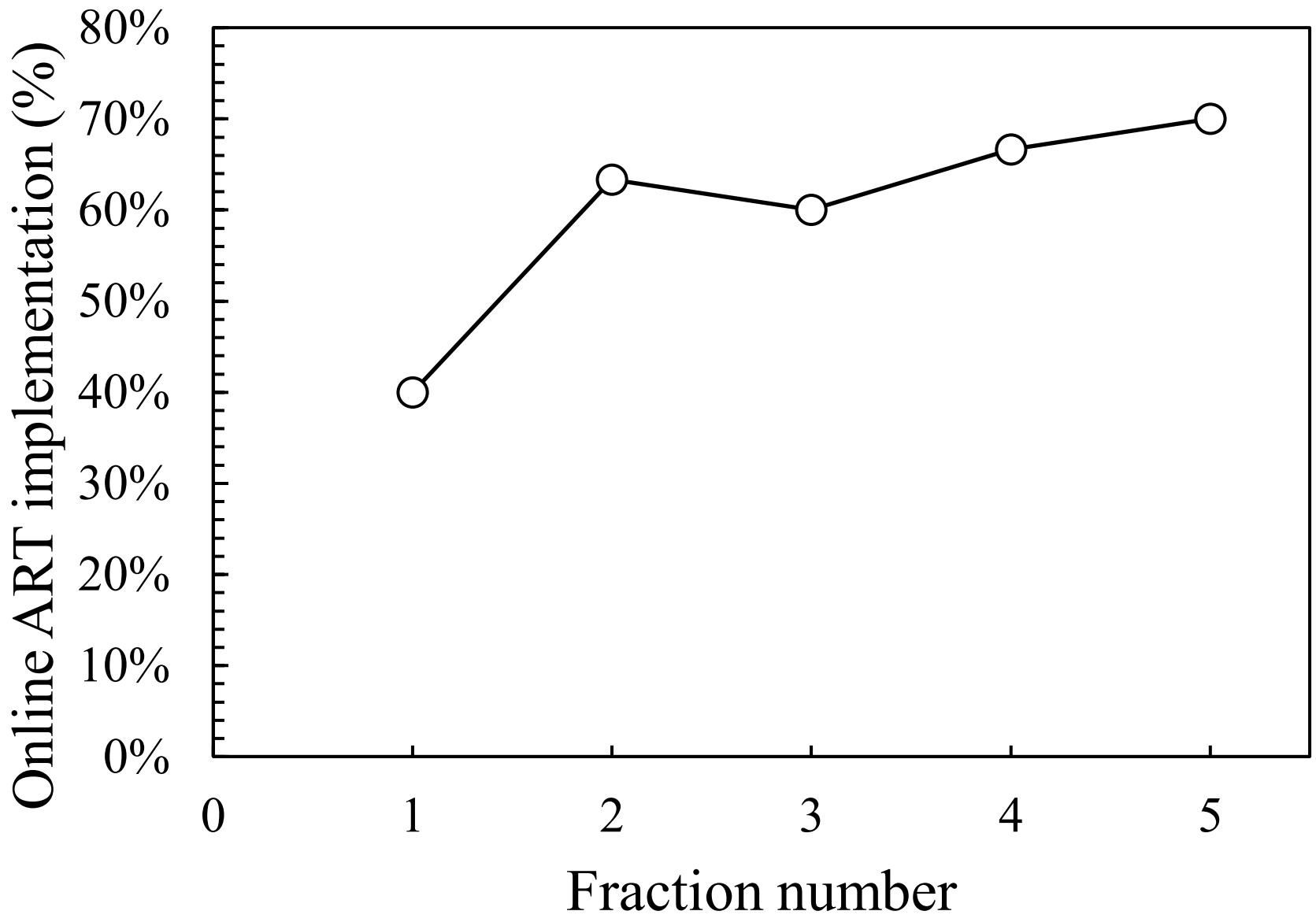

Figure. S1. The percentage of online dose re-optimization implementation in each treatment fraction (n = 30). The implementation of online dose re-optimization increased from 40.0% in the first fraction to 70.0% in the fifth fraction.

(a)

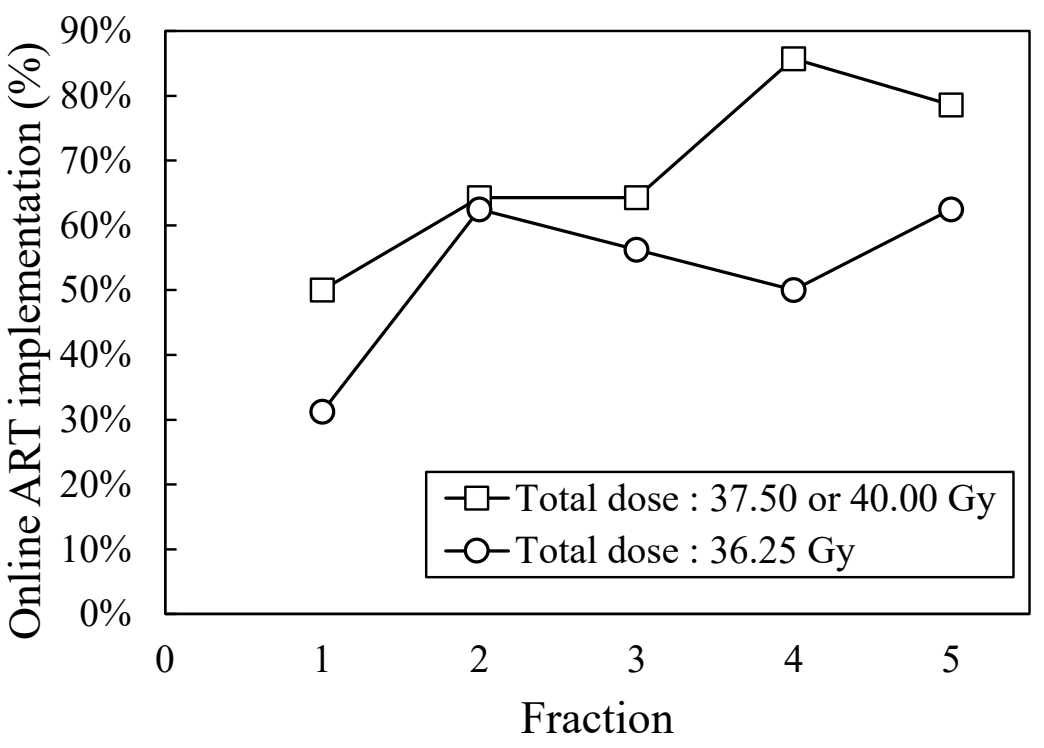

(b)

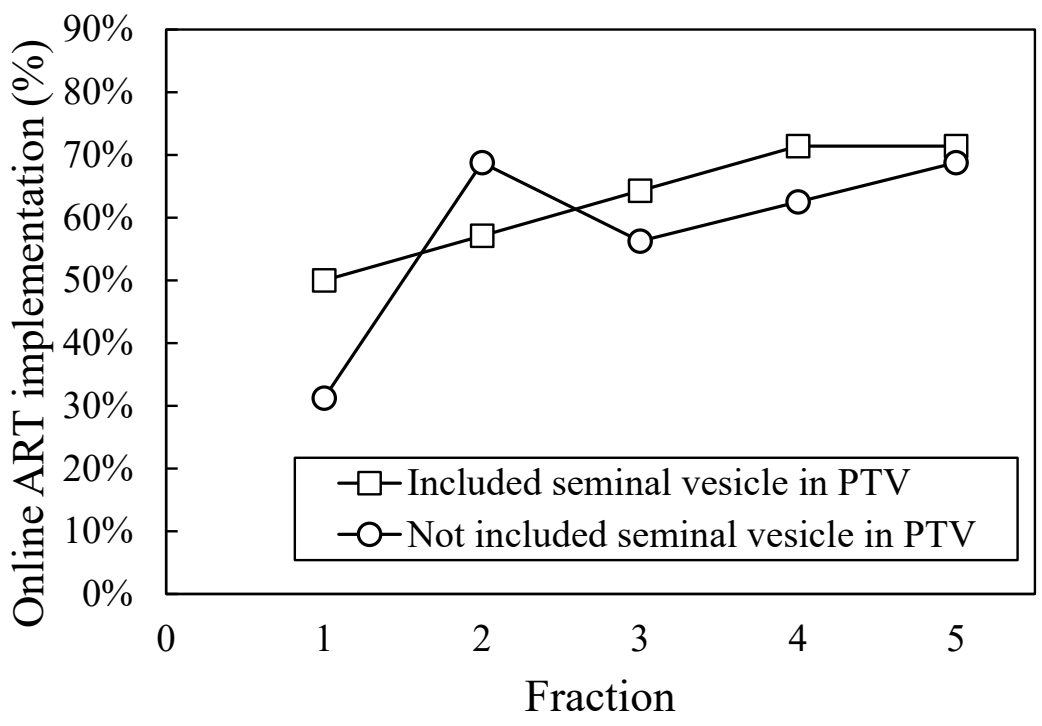

(c)

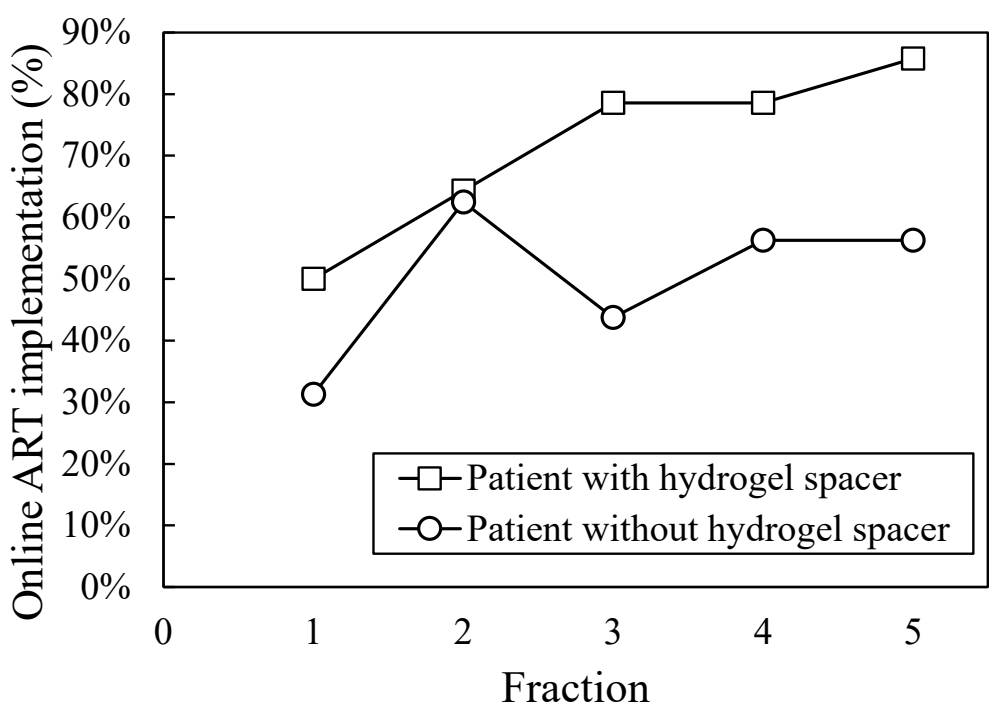

Figure. S2. The online ART implementation according to (a) prescribed dose, (b) the inclusion of the seminal vesicle in PTV, and (c) the insertion of a hydrogel spacer.

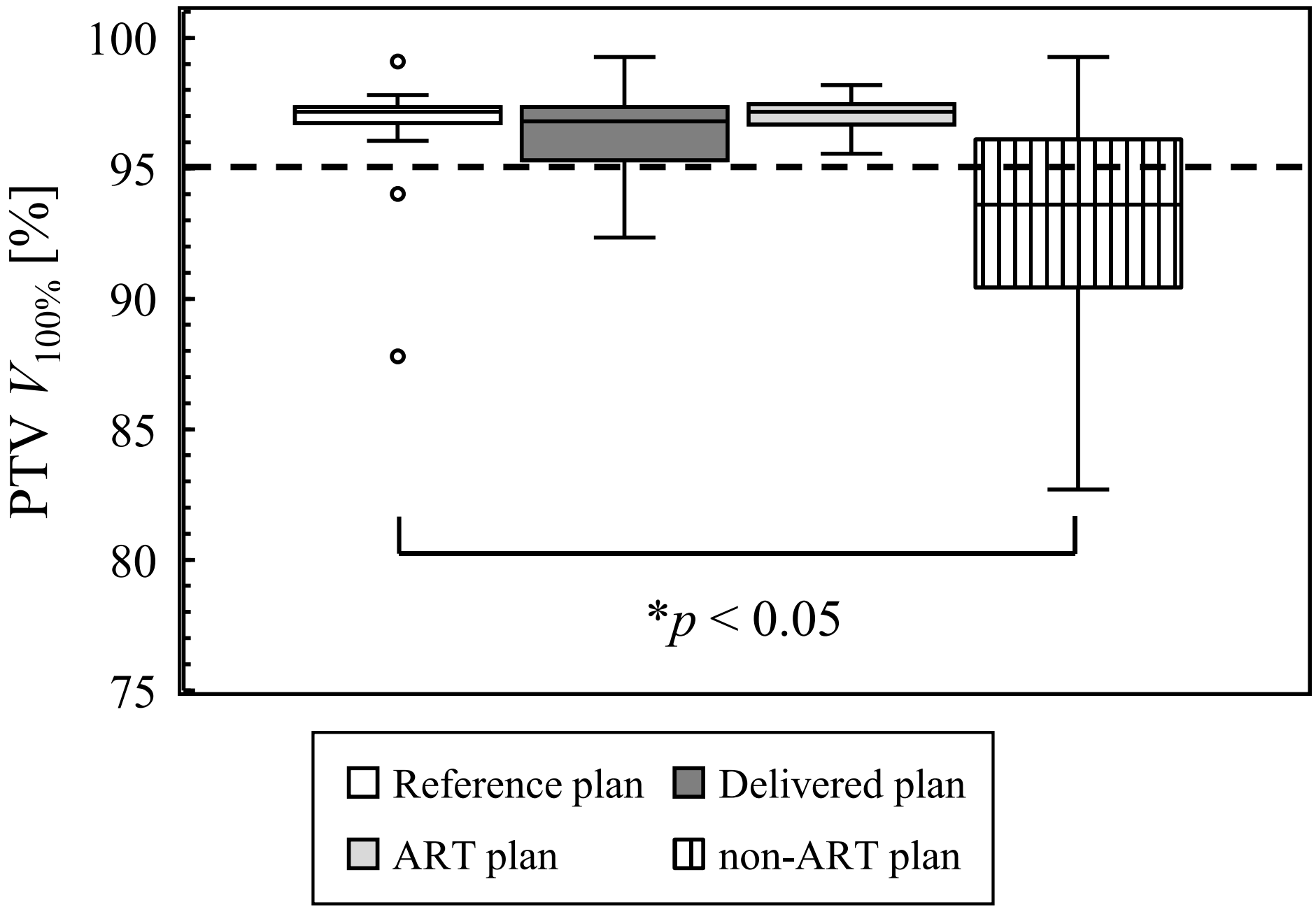

Figure. S3. Boxplot comparison of dose-volume parameters for PTV $V_{100\%}$ for the reference plan, delivered plan, ART plan and non-ART plan, respectively. The dashed line shows the dose constraint [10].

(a)

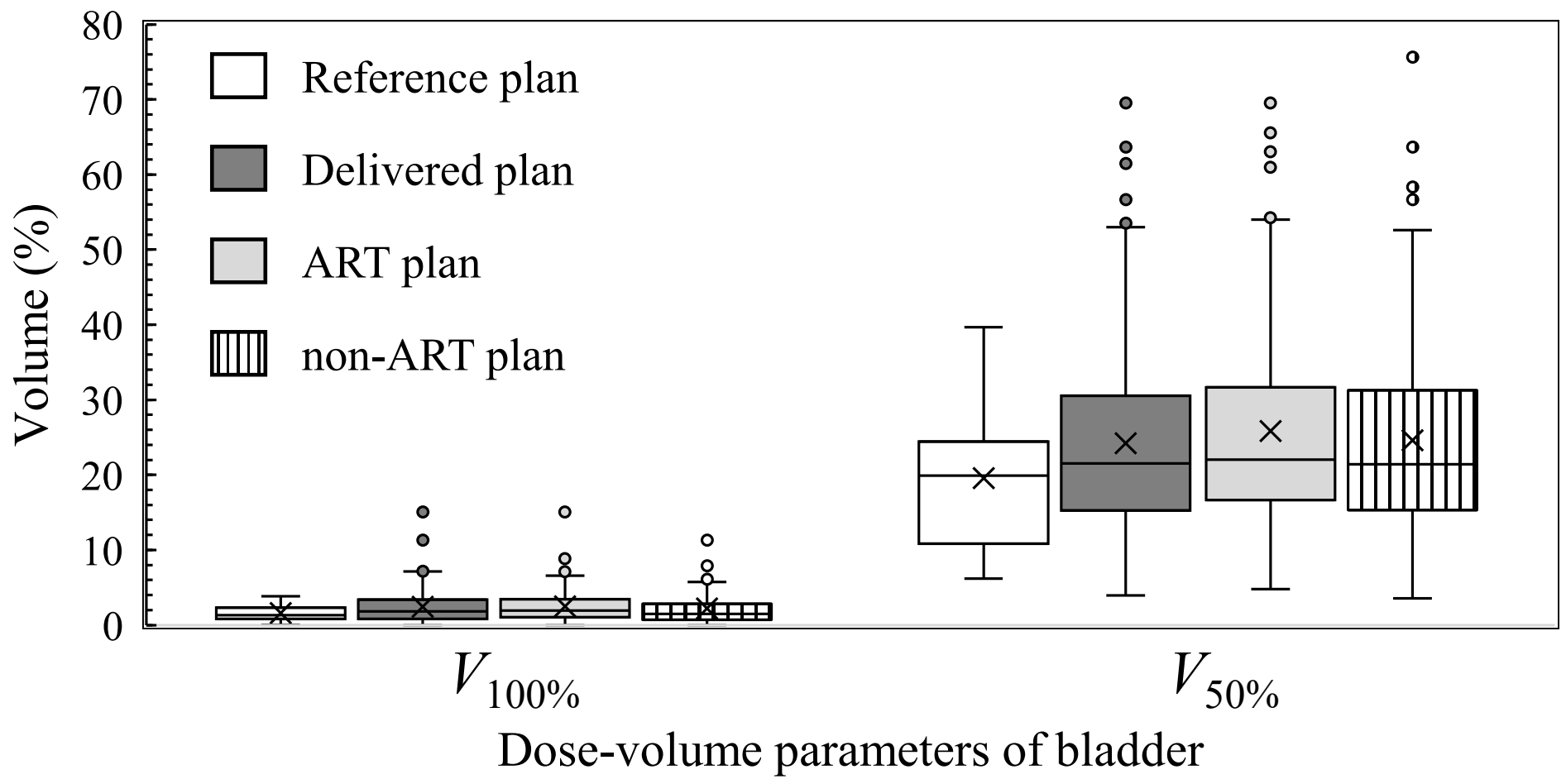

Reference plan
Delivered plan
ART plan
non-ART plan
Volume (%)
0
10
20
30
40
50
60
70
80
$V_{100\%}$
$V_{50\%}$
Dose-volume parameters of bladder

(b)

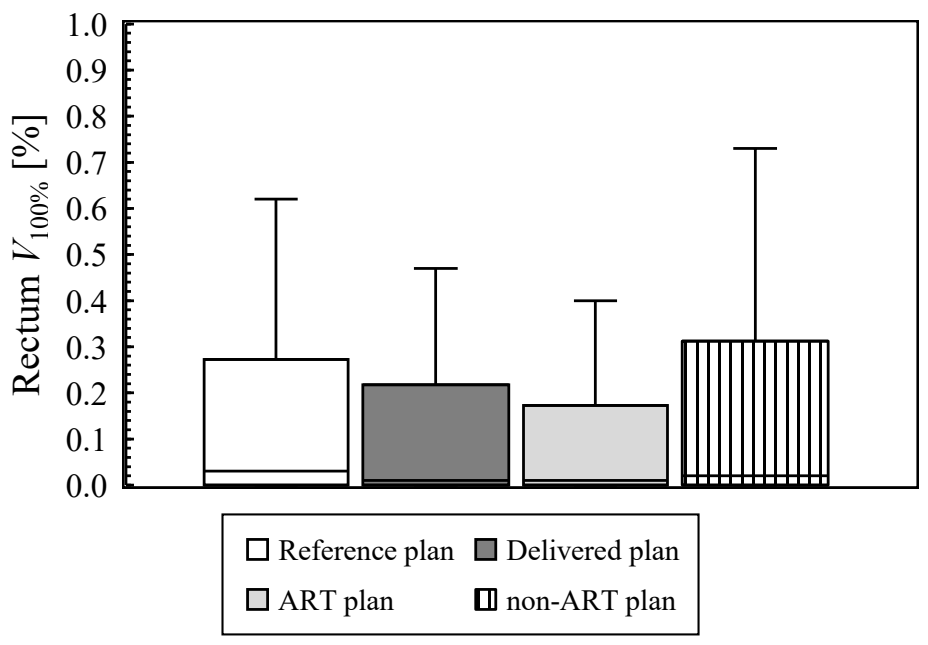

Rectum $V_{100\%}$ [%]
0.0
0.1
0.2
0.3
0.4
0.5
0.6
0.7
0.8
0.9
1.0
Reference plan
Delivered plan
ART plan
non-ART plan

(c)

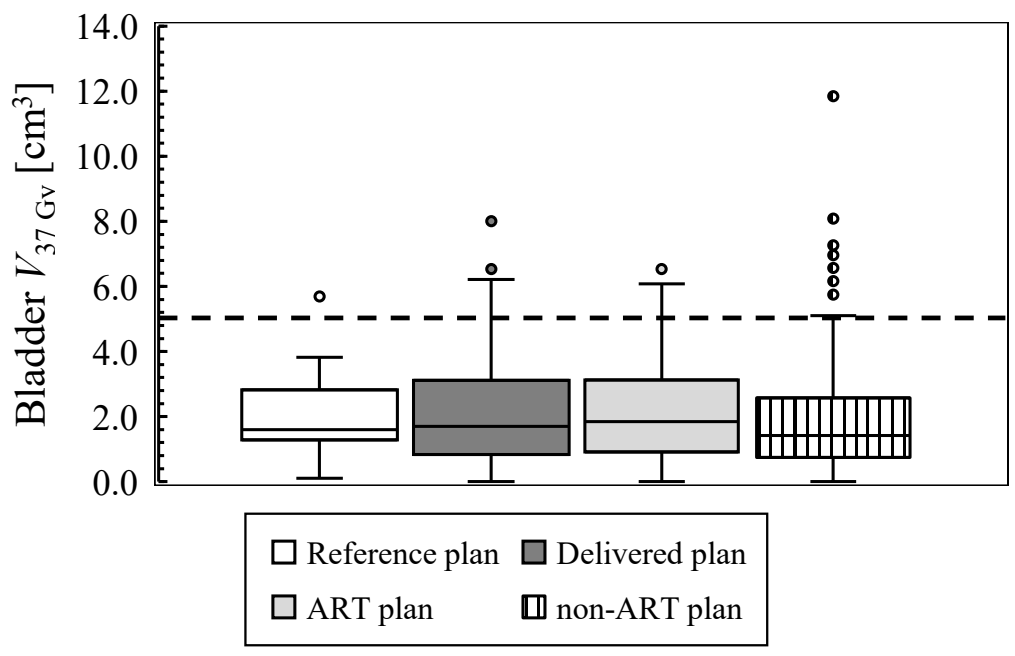

Bladder $V_{37\ Gy}$ [cm^3]
0.0
2.0
4.0
6.0
8.0
10.0
12.0
14.0
Reference plan
Delivered plan
ART plan
non-ART plan

(d)

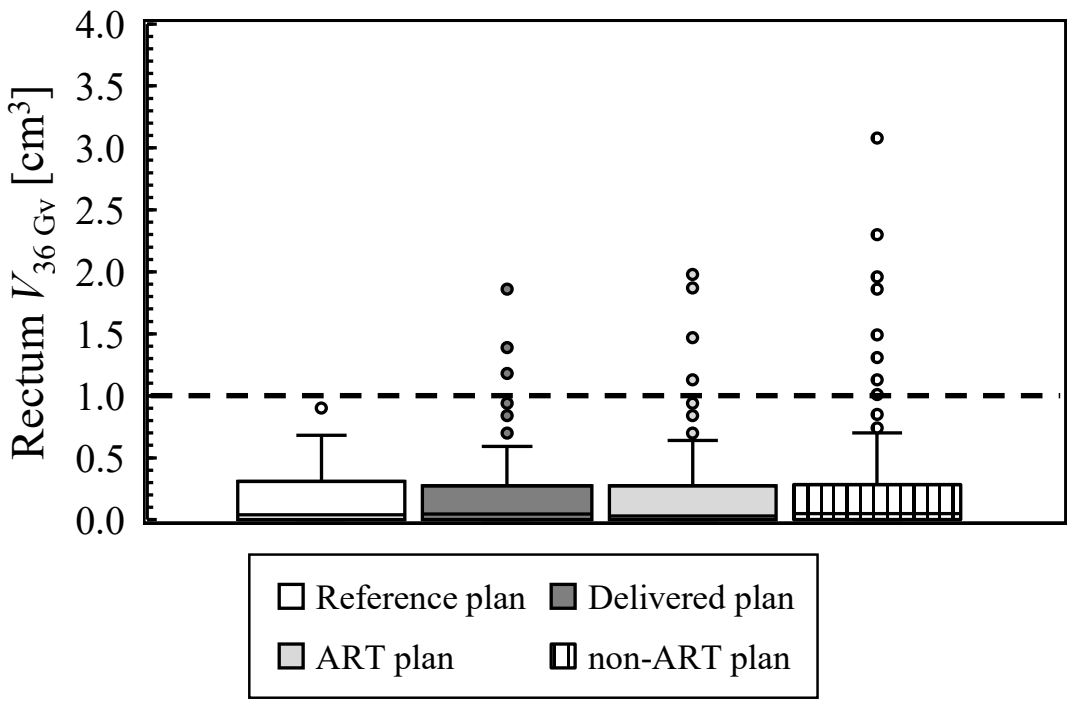

Rectum $V_{36\ Gy}$ [cm^3]
0.0
0.5
1.0
1.5
2.0
2.5
3.0
3.5
4.0
Reference plan
Delivered plan
ART plan
non-ART plan

(e)

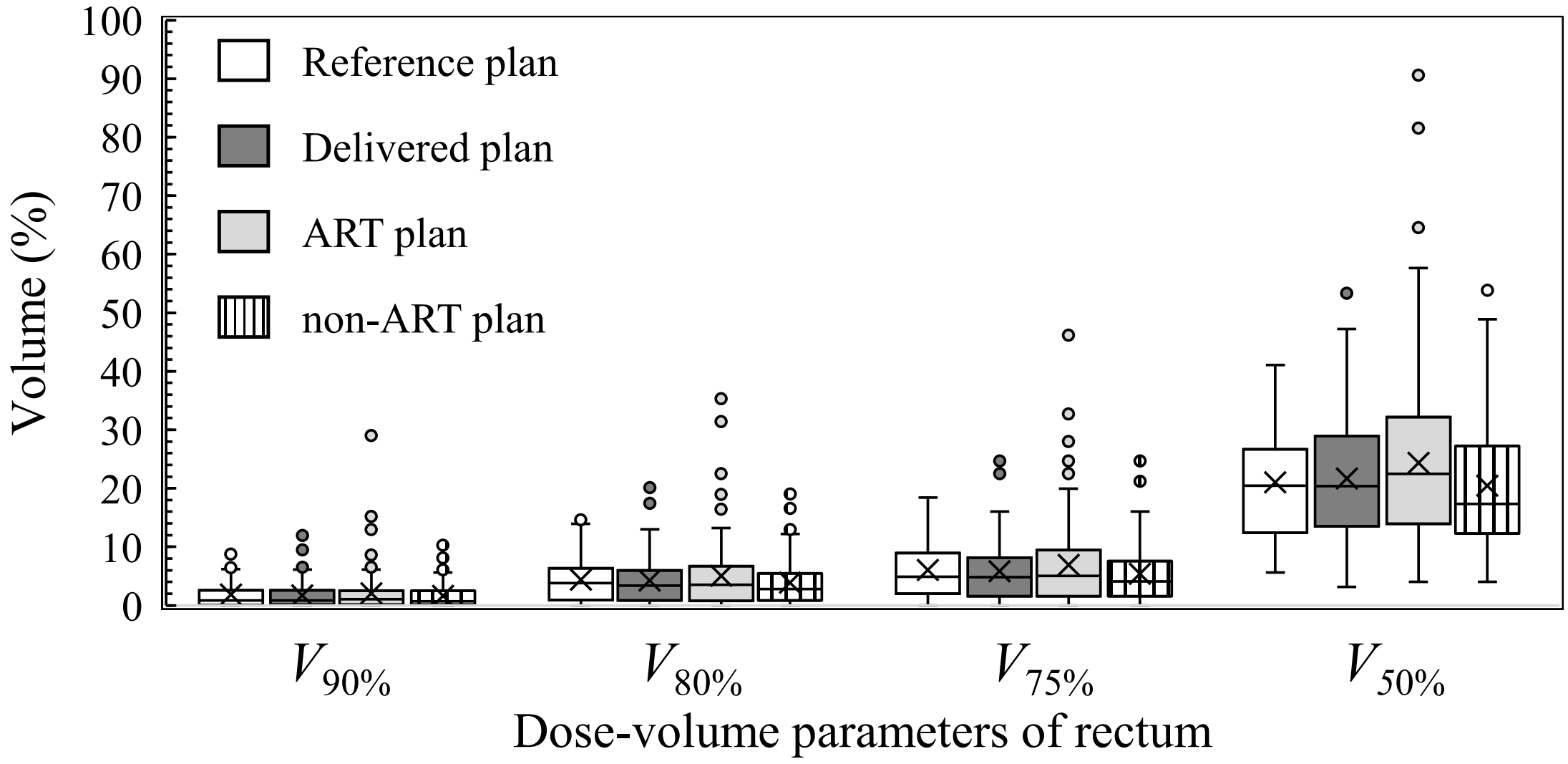

Figure. S4. Boxplot comparison of dose-volume parameters for (a) Bladder $V_{100\%}$ and $V_{50\%}$, (b) Rectum $V_{100\%}$, (c) Bladder $V_{37\ Gy}$, (d) Rectum $V_{36\ Gy}$, and (e) Rectum $V_{90\%}$, $V_{80\%}$, $V_{75\%}$, and $V_{50\%}$ between the reference plan, delivered plan, ART plan and non-ART plan, respectively. The dashed line shows the dose constraint [10].

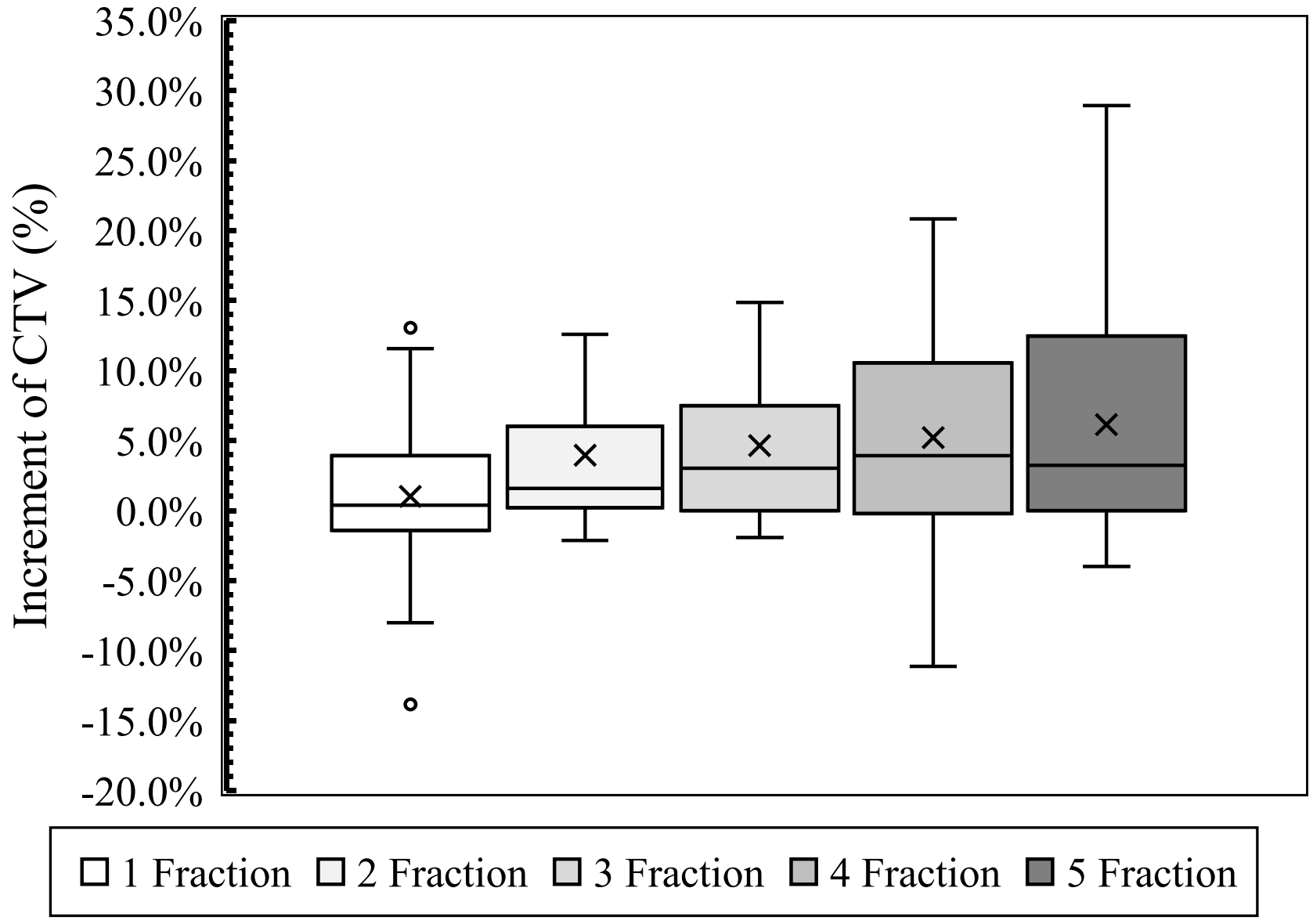

Figure. S5. Increment of CTV from reference plan.
